# TNF-induced metalloproteinase-9 production is associated with neurological manifestations in HTLV-1-infected individuals

**DOI:** 10.3389/fimmu.2022.954103

**Published:** 2022-10-13

**Authors:** Mariele Guerra, Natália B. Carvalho, Silvane Santos, Mauricio T. Nascimento, Renata Sá, Augusto M. Carvalho, Edgar M. Carvalho, Lucas P. Carvalho

**Affiliations:** ^1^ Immunology Service, University Hospital Complex Professor Edgard Santos (C-HUPES), Federal University of Bahia (UFBA), Salvador, Bahia, Brazil; ^2^ Biology Department, State University of Feira de Santana, Feira de Santana, Bahia, Brazil; ^3^ Gonçalo Moniz Institute (IGM), Oswaldo Cruz Foundation Fundação Oswaldo Cruz (FIOCRUZ), Salvador, Bahia, Brazil; ^4^ National Institute of Science and Technology - Tropical Diseases Conselho Nacional de Pesquisa/Ministério da Ciência e Tecnologia (CNPq/MCT), Salvador, Bahia, Brazil

**Keywords:** HTLV-1, HAM/TSP, metalloproteinases, TIMPs, inflammation

## Abstract

HTLV-1-infected individuals may develop a neurologic inflammatory condition known as HTLV-1-associated myelopathy (HAM/TSP), in which the high production of TNF is observed. These patients exhibit higher proviral loads, enhanced production of proinflammatory cytokines and lymphocyte proliferation in comparison to asymptomatic HTLV-1 carriers and those presenting overactive bladder (OAB-HTLV-infected). Metalloproteinases (MMPs) are known to degrade the components of the blood-brain barrier, favoring the migration of infected cells into the central nervous system. Moreover, the unbalanced production of MMPs and their inhibitors (TIMPs) has also been associated with tissue damage. The present work studied the production of MMP-9 and TIMPs in HTLV-1-infected individuals with and without neurological manifestations. HAM/TSP patients presented higher concentrations of MMP-9 in peripheral blood mononuclear cell (PBMC) culture supernatants, as well as a higher MMP-9/TIMP-3 ratio when compared to the other groups studied. MMP-9 levels positively correlated with proviral load and TNF in OAB-HTLV-infected individuals, and the *in vitro* neutralization of TNF significantly decreased MMP-9 levels in PBMC culture supernatants. Our findings indicate an association between MMP-9 production and the proinflammatory state associated with HTLV-1 infection, as well as HAM/TSP.

## Introduction

Approximately 10 million people are infected with HTLV-1 worldwide (1). Although most HTLV-1-infected individuals remain asymptomatic, some will develop neurological disorders, such as overactive bladder (OAB) and HTLV-1-associated myelopathy/tropical spastic paraparesis (HAM/TSP) (2–4). HAM/TSP is an inflammatory condition in which the principal neuropathological finding is chronic myelitis, characterized by parenchymal infiltration consisting mainly of lymphocytes (2–5). The mechanisms driving the development of HAM/TSP in HTLV-infected individuals are not well-understood. OAB, a urologic manifestation, is characterized by increased urgency and frequency, as well as urinary loss (6–8). Although asymptomatic HTLV-1 carriers present urodynamic alterations, dysuria and urinary loss are more frequent among patients with severe HAM/TSP (9). Thus, it has been proposed that OAB may be an oligosymptomatic presentation of myelopathy, or an initial manifestation of HAM/TSP (10).

The host immune response against HTLV-1 is characterized by increased lymphocyte proliferation followed by exacerbated production of proinflammatory cytokines and chemokines, such as IFN-γ, TNF, CXCL-10. Patients with HAM/TSP produce higher levels of these molecules, and also present higher proviral loads compared to asymptomatic HTLV-1 carriers (11–15). TNF and CXCL-10 have been detected in cerebrospinal fluid (CSF), suggesting the potential for the virus to cross the blood-brain barrier (4, 16–19). The blood-brain barrier is composed of cells (astrocytes, pericytes, neurons and endothelial cells), while the extracellular matrix is formed by proteins, such as fibers, collagen, elastin, laminin and fibronectin (16, 17, 20). The latter is responsible for permitting the entry of ions, molecules and cells into the central nervous system. However, in the context of pathologies, increased permeability of blood-brain barrier facilitates the entry of pathogens and infected cells into the central nervous system (16, 17).

Metalloproteinases (MMPs) are molecules that degrade extracellular matrix components. Unbalanced production between MMPs and their inhibitors (TIMPs) has been associated with tissue damage in several inflammatory conditions, including arthritis, cutaneous leishmaniasis, cancer and cardiovascular diseases (21–23). Among the MMPs, MMP-3 and -9 have been the focus of studies on HTLV due to their ability to degrade Types IV and V collagen, fibronectins and laminin, all components of the basement membrane in the blood-brain barrier (24–27). It has been documented that, when activated, astrocytes in the central nervous system produce pro-inflammatory cytokines, contributing to increased permeability in the blood-brain barrier (28). Moreover, astrocytes are also known to produce MMP-9 when in contact with HTLV-infected T cells (29). Moreover, MMP-9 has been evidenced in CSF, and MMP-9 production in the central nervous system has been hypothesized to be associated with HAM/TSP development (25, 29, 30). The literature contains several studies investigating imbalances in MMP and TIMP levels in serum and CSF in association with immunopathology in HTLV (24–26). However, since HTLV primarily infects T cell populations, we chose to focus on the production of MMP-9 and its TIMP-3 inhibitor by peripheral blood mononuclear cells (PBMC) in asymptomatic HTLV-1 carriers, HTLV-1-infected individuals with OAB (HTLV-1-OAB), and HAM/TSP patients. We found a relevant association between HAM/TSP and high levels of MMP-9, in addition to low levels TIMP-3. Moreover, TNF was identified as a key cytokine linked to MMP-9 production by PBMCs in HTLV-1-infected patients regardless of myelopathy.

## Material and methods

### Patients

The present study included 120 participants followed at the HTLV-1 multidisciplinary outpatient clinic at the Professor Edgard Santos University Hospital Complex of the Federal University of Bahia (HUPES-UFBA), Salvador, Bahia-Brazil. Subject participation was strictly voluntary, all individuals provided written informed consent and the present research protocol was submitted to and approved by the Institutional Review Board of the Federal University of Bahia. HTLV-1 infection was diagnosed through the detection of antibodies by ELISA (Cambridge Biotech Corp., Worcester, MA, USA) and subsequently confirmed by Western blot (HTLV blot 2.4, Genelab, Singapore). Participants were classified into three groups: i) HTLV-1 carriers (asymptomatic HTLV-1-infected individuals), ii) HTLV-1-OAB (HTLV-1-infected individuals with urinary manifestations indicative of neurogenic bladder, i.e., urgency or other urinary symptoms, such as nocturia and incontinence), iii) HAM/TSP (HTLV-1-infected individuals classified as definite HAM/TSP according to the de Castro-Costa diagnostic criteria (31). HTLV-1-infected individuals aged between 23-75 years of both genders participated in the study. Individuals coinfected with HIV, hepatitis virus (B or C), syphilis, and those using immunosuppressive drugs, or who were pregnant, were not recruited for this study. None of the studied patients were using corticosteroids.

### Proviral load determination

DNA was extracted from 10^6^ PBMCs using proteinase K and salting-out method. HTLV-1 proviral load was quantified using a real-time TaqMan PCR method on an ABI Prism 7700 Sequence detector system (Applied Biosystems) (32). Five plasmid dilution points were used to calculate the standard curve (pcHTLV-ALB). All samples were analyzed in duplicate, both for the quantification of HTLV-1 proviral load and albumin levels. Albumin DNA was used as an endogenous reference. Normalized HTLV-1 proviral load values were calculated using the ratio (HTLV-1 DNA average copy number/albumin DNA average copy number) × 2×10^6^, and expressed as the number of HTLV-1 copies per 10^6^ PBMCs.

### Peripheral blood mononuclear cell cultures

Peripheral blood mononuclear cells were isolated from heparinized venous blood by Ficoll-Paque (GE Healthcare, Chicago, IL, USA) gradient centrifugation. After washing in saline, cell concentrations were adjusted to 3×10^6^ cells in 1 ml of RPMI-1640 medium (Thermofisher Scientific, NY, USA) supplemented with 10% FBS (Thermofisher Scientific, NY, USA), penicillin (100 U/mL) and streptomycin (100 µg/mL). PBMCs were dispensed into 24-well plates and incubated at 37°C under 5% CO_2_ for 72 hours. Supernatants were collected from PBMCs cultures and stored at -70°C until the time of MMP-9, TIMP-3 and TNF quantification using an ELISA KIT (R&D Systems, Minneapolis, MS, USA), in accordance with manufacturer instructions. To investigate the effects of cytokine blockade on MMP-9 production, anti-TNF, anti-IFN-γ, anti-IL-1β anti-IL-6 and anti-TGFβ (R&D Systems, Minneapolis, MS, USA) monoclonal antibodies were added to some PBMC cultures at a concentration of 10 μg/mL. Results are expressed in pg/mL.

### Statistical analysis

Differences between groups were analyzed using the Mann-Whitney U test (for comparisons between two independent groups). Receiver operator characteristics (ROC) curve analysis was used to evaluate the ability of MMP-9 levels to distinguish between asymptomatic HTLV-1 carriers, HTLV-1-OAB individuals and HAM/TSP patients. Spearman’s rank correlation was employed to test correlations. All data were analyzed using GraphPad Prism 5.01 (GraphPad Software, San Diego, CA, USA). Differences were considered statistically significant when p value ≤ 0.05.

## Results

### Patients, proviral load and inflammatory response

The present study aimed to quantify MMP-9 and TIMP-3 production by PBMCs in HTLV-1-infected individuals with or without OAB, as well as in HAM/TSP patients. We enrolled 40 HTLV-1-infected asymptomatic individuals, 40 HTLV-1-OAB and 40 HAM/TSP patients. The studied groups did not differ with regards to sex or age (**Table 1**). Consistent with previous reports, proviral loads in HAM/TSP patients were significantly higher than in HTLV-1 asymptomatic carriers or HTLV-1-OAB patients (**Table 1** and **Supplementary Figure 1A**) (12, 13). Also, as was previously documented, significantly higher spontaneous production of TNF was observed in PBMC culture supernatants from HAM/TSP patients compared to HTLV-1 carriers or HTLV-1-OAB patients (**Table 1** and **Supplementary Figure 1B**) (12, 14, 16).

**Table 1:** Clinical and demographical characteristics of studied groups.

	Asymptomatic (n=40)	OAB-HTLV (n=40)	HAM/TSP (n=40)	P value
**Female**	27 (67%)	28 (70%)	29 (72%)	0,74*
**Age (years)**	50 (25-69)	55 (25-71)	55 (23-75)	0,33**
**Viral load(copies/10^6^ cells)**	31085 (0-757662)	111275 (0-992164)	150622 (0-1768442)	<0.05**^a^
**TNF (pg/ml)**	99 (0-3956)	504 (0-3956)	1043 (0-4880)	<0.05**^a^

*Fisher test (χ^2^).

**Mann Whitney test.

a Differences between HAM/TSP and other groups.

### Unbalanced production of MMP9 and TIMP3 in HAM/TSP patients

**Figure 1:**
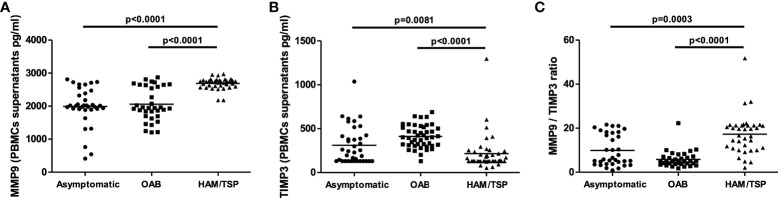
*HAM/TSP patients present increased MMP-9 levels in PBMC culture supernatants.* MMP-9 **(A)** and TIMP-3 **(B)** levels in PBMC supernatants from HTLV-1 carriers, OAB-HTLV-infected and HAM/TSP patients, as assayed by ELISA. **(C)** MMP-9/TIMP-3 ratio. Bars represent median values from each group. Nonparametric testing (Mann-Whitney) was used to compare groups.

**Figure 2:**
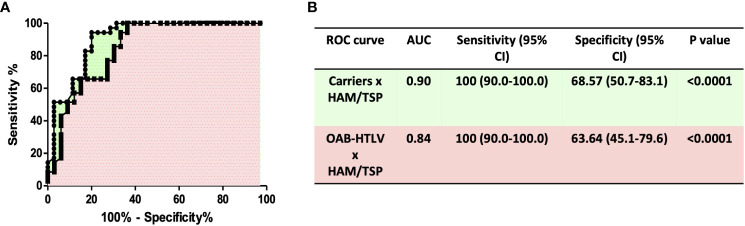
*ROC curve analysis of MMP-9 levels distinguishes HAM/TSP patients from HTLV-1 carriers and OAB-HTLV-infected individuals*. **(A)** ROC curves were built based on MMP-9 levels in PBMC supernatants from HTLV-1 carriers, OAB-HTLV-infected and HAM/TSP patients. **(B)** ROC curve sensitivity and specificity estimations with 95% confidence intervals (CI), as well as Area Under Curve (AUC) values, corresponding P values, and selected cut-off values.

HAM/TSP development in HTLV-infected patients has been associated with the infiltration of leukocytes into the central nervous system. Several studies have documented the association of MMP-9 production in CSF with blood-brain barrier disruption (33, 34). Therefore, we assessed the concentrations of MMP-9 and TIMP-3 in PBMC culture supernatants from HTLV-1-infected individuals. MMP-9 levels were found to be significantly higher in PBMC cultures from HAM/TSP patients compared to HTLV-1 carriers and HTLV-1-OAB individuals (**Figure 1A**). MMP-9 activity is inhibited by TIMPs (22, 26, 35). Moreover, lower TIMP-3 levels were observed in HAM/TSP patients compared to the other HTLV-1-infected individuals (**Figure 1B**). Finally, the unbalanced production of MMP-9 and TIMP-3 was evidenced in HAM/TSP patients *via* higher MMP-9/TIMP3 ratios compared to HTLV-1 carriers and HTLV-1-OAB individuals (**Figure 1C**). ROC analysis confirmed that the ability of MMP-9 to distinguish between HAM/TSP patients and asymptomatic HTLV-1 carriers, as well as HTLV-1-OAB patients, with high accuracy (**Figures 2A, B**).

### MMP-9 levels correlate with inflammatory response and proviral load in OAB-HTLV-1 patients

**Figure 3:**
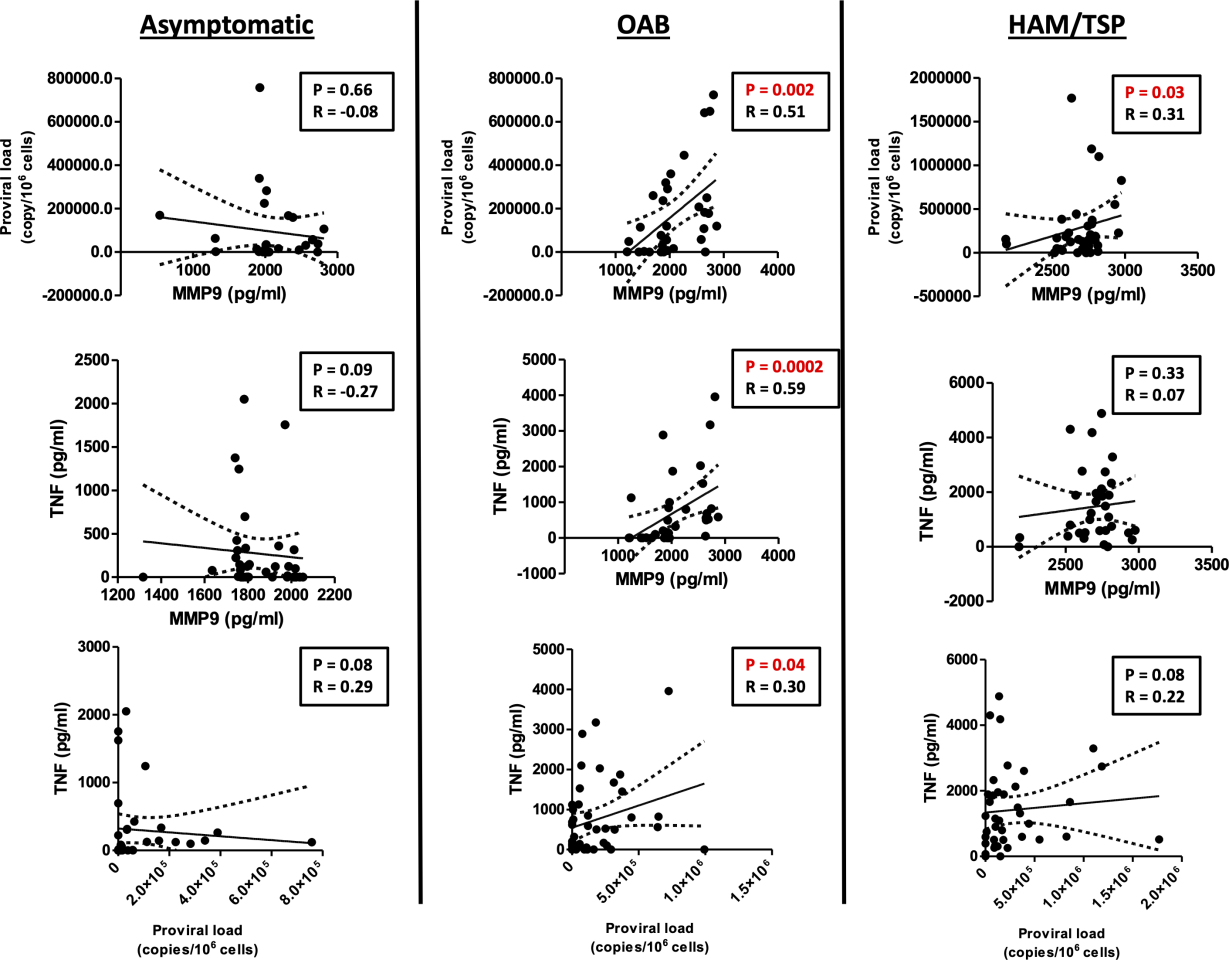
*MMP-9 levels positively correlate with proviral load and TNF in OAB-HLTV-1-infected patients.* Correlation between MMP-9 levels and proviral load, as well as TNF, in asymptomatic HTLV-1-infected individuals, OAB-HTLV-1-infected and HAM/TSP patients. Spearman’s correlation rank testing was used for comparisons.

Proviral load has been associated with an exacerbated inflammatory response and HAM/TSP development (12, 14). To study the association between MMP-9 production and neurological manifestations associated with HTLV-1 infection, we investigated whether MMP-9 levels correlated with proviral load and TNF production. Our results showed a positive correlation between MMP-9 production with proviral load and TNF production in OAB patients, indicating the association of MMP-9 with the immunopathology and neurological manifestation (**Figure 3**).

### TNF induces MMP-9 production in HTLV

**Figure 4:**
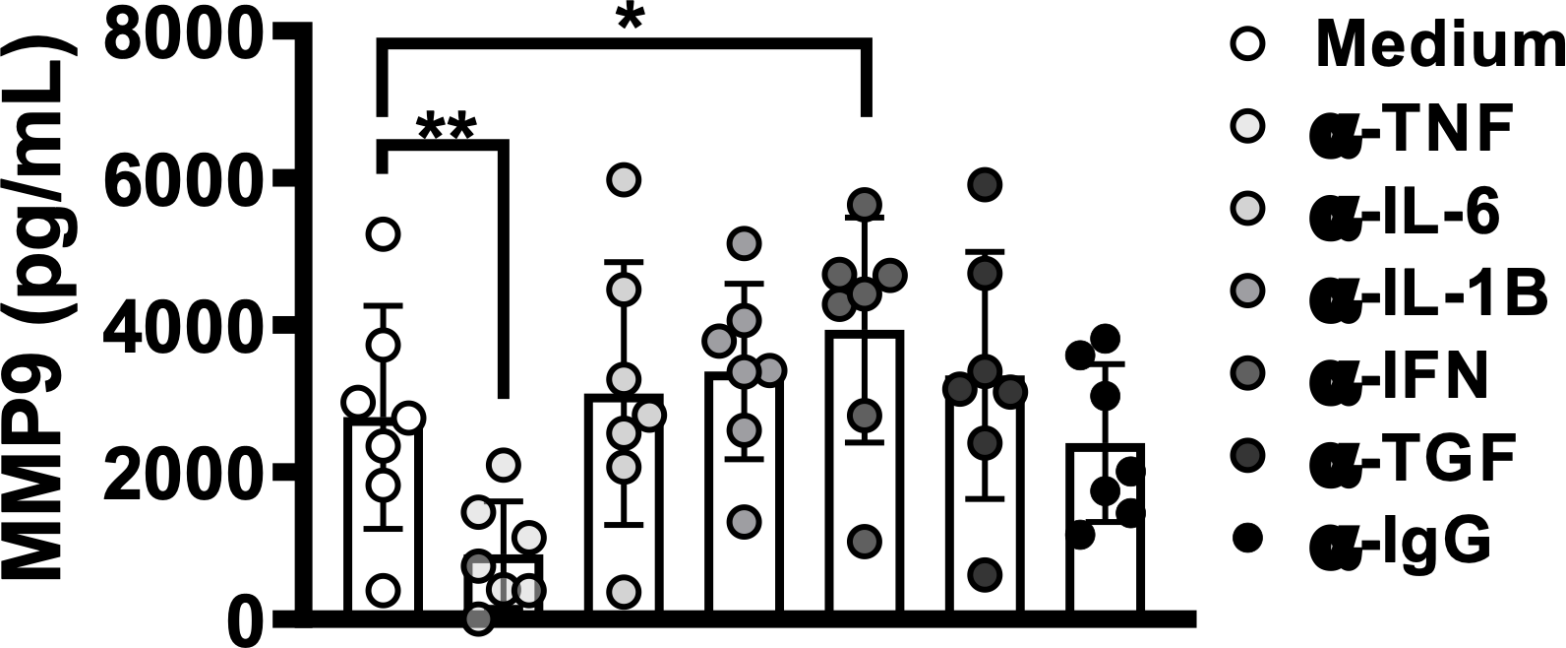
*TNF blockade decreases MMP-9 levels in HTLV-1 infection*. PBMCs from HTLV-1-infected individuals, including those with or without OAB or HAM/TSP, were cultured for 72 hours in the presence or absence of the following monoclonal antibodies: anti-TNF, -IFN-γ, -IL-1-β, -IL-6 and -TGF-β. MMP-9 levels were assessed in culture supernatants by ELISA. Bars represent median values from each group. Nonparametric testing (Mann-Whitney) was used to compare among groups. *P < 0.05, **P < 0.005.

The present results indicate that HAM/TSP patients produce higher levels of TNF compared to asymptomatic or OAB-HTLV-infected individuals. Furthermore, it has been documented that TNF induces MMP-9 production (36, 37). To investigate the effect of TNF levels on MMP-9 production in HTLV-1, we cultured PBMCs from HTLV-1-infected individuals in the presence of anti-TNF antibodies. The neutralization of TNF decreased the spontaneous production of MMP-9 in PBMC culture supernatants (**Figure 4**). Other cytokines, such as IFN-γ, IL-1-β, IL-6 and TGF-β, are also known to affect MMP-9 production (38, 39). Our results indicate a protective role for IFN- γ, since the neutralization of this cytokine subsequently increased MMP-9 production in PBMC culture supernatants (**Figure 4**). Together, these findings demonstrate that proviral load influences proinflammatory response, which is linked to both the production of MMP-9 and HAM/TSP development.

## Discussion

Patients with HAM/TSP present high proviral load, and HTLV-1-infected T cells from these individuals produce high levels of proinflammatory mediators, e.g., TNF and CXCL-10, when compared to asymptomatic carriers or OAB-HTLV-1-infected individuals (14). The development of HAM/TSP is associated with the migration of T cells across the blood-brain barrier, composed of endothelial cells that selectively coordinate cell migration (5, 16, 17). Thus, increased permeability in the blood-brain barrier allows for the infiltration of inflammatory cells into the central nervous system (16, 17). Under such conditions, MMPs can actively damage the blood-brain barrier, and MMP-9 has been shown to be an important mediator in this process (40). For instance, increased levels of MMP-9 are found after stroke (33, 34). MMP-9 is mainly secreted by neutrophils, mononuclear phagocytes and fibroblasts (35, 41). Since the presently performed experiments employed PBMCs, it is likely that monocytes are the main source of MMP-9. However, other cell types also contribute to the production of MMP-9, as it has been shown that HTLV-1-infected CD4+ T cells induce astrocytes to produce MMP-9, and another study demonstrated that HTLV-1-infected cells are prone to produce MMP-9 through the transactivation of its gene by the viral Tax protein (29, 42). Our data indicate the low production of TIMP-3 in HAM/TSP patients. Imbalance in the production of MMPs/TIMPs can lead to excessive degradation of the extracellular matrix, as well as changes in the interconnectivity of the cells that make up the blood brain barrier (25). Thus, alterations in the balance of MMP/TIMP production appear to be relevant in neurological diseases mediated by T lymphocytes. Additionally, we found that MMP-9 levels correlated positively with proviral load, which supports the hypothesis that MMP-9 participates in a deleterious inflammatory response.

TNF, a proinflammatory cytokine produced during viral infection, can stimulate monocytes to secrete MMPs (23, 25). In HTLV-infected individuals, it has been demonstrated that HTLV-infected cells produce TNF and other proinflammatory cytokines/chemokines through the nuclear translocation of NF kappa B components, mediated by the viral Tax protein (43). Our data show that the HAM/TSP patients studied herein produce more TNF than asymptomatic carriers or OAB-HTLV-infected patients, suggesting the contribution of this cytokine to the immunopathogenesis of HAM/TSP. Moreover, our results also show that MMP-9 production in HTLV1-infected patients is partially dependent on TNF. We further identified a strong positive correlation between MMP-9 and TNF in OAB individuals, despite the lack of such a correlation in patients with HAM/TSP. It is possible that the similar MMP-9 levels found among the HAM/TSP individuals, in contrast to OAB, contributed to the lack of a correlation between MMP-9 and TNF in this group. Furthermore, as most HAM/TSP patients present high proviral load, it is known that the viral Tax protein directly induces MMP-9 production, which may abrogate the effects of TNF in HAM/TSP patients (44). The use of etanercept, a TNF inhibitor, has been shown to reduce MMP-9 levels in children with polyarticular juvenile idiopathic arthritis, corroborating the role of TNF in MMP-9 production (45). Although TNF possesses antiviral properties, in the context of HTLV infection, its production does not seem to contribute to viral killing, as TNF has been observed to positively correlate with proviral load (46–48). Further, HTLV-1-infected individuals treated with etanercept did not present any worsening of HTLV-associated T cell leukemia over a five-year period (49). Altogether, these results support the notion that HTLV-infected individuals may benefit from treatments designed to attenuate TNF production.

Some works have reported increased levels of MMP-9 in the central nervous system in individuals with HAM/TSP (26, 50, 51). However, our findings also indicate a strong correlation between TNF and MMP-9 production in OAB-HTLV-infected patients. We were able to clearly identify two groups of MMP-9 producers (high and low producers) among the asymptomatic and OAB individuals studied. OAB individuals presenting neurological manifestations are believed to eventually progress to HAM/TSP.

The present results were achieved *via* a cross-sectional study, which presents limitations. As HAM/TSP is an insidious complication of HTLV infection, long-term cohort studies are necessary to definitively validate the link between TNF and MMP-9 production in the context of HAM/TSP development. In conclusion, our findings enhance the data available in the literature by shedding light on the association between unbalanced production of MMP-9 and TIMP-3 by PBMCs and the development of HAM/TSP, and also serve to highlight the important role of TNF in triggering pathologic responses in HTLV infection. The identification of pathways involved in the immunopathogenesis of HTLV-1-associated diseases may aid in the quest to discover novel therapeutic approaches.

## Data availability statement

The original contributions presented in the study are included in the article/**Supplementary Materials**. Further inquiries can be directed to the corresponding author.

## Ethics statement

The studies involving human participants were reviewed and approved by School of Medicine, Federal University of Bahia. The patients/participants provided their written informed consent to participate in this study.

## Author contributions

MG, NC, SS, EC, and LC participated in the study design. MG, NC, SS, AC, EC, and LC drafted the manuscript. MG, MN, NC, and RS performed the experiments. All authors contributed to the article and approved the submitted version.

## Funding

FAPESB and CNPq - Instituto Nacional de Ciências e Tecnologia para Doenças Tropicais (INCT-DT) Project number: 573839/2008-5 – INCT-DT.

## Acknowledgments

We thank the staff of HTLV-1 clinic at the Professor Edgard Santos University Hospital Complex (HUPES-UFBA). The authors would like to thank Andris K. Walter for critical analysis, English language revision and manuscript copyediting assistance.

## Conflict of interest

The authors declare that the research was conducted in the absence of any commercial or financial relationships that could be construed as a potential conflict of interest.

## Publisher’s note

All claims expressed in this article are solely those of the authors and do not necessarily represent those of their affiliated organizations, or those of the publisher, the editors and the reviewers. Any product that may be evaluated in this article, or claim that may be made by its manufacturer, is not guaranteed or endorsed by the publisher.

## Supplementary material

Supplementary Figure 1
*Increased proviral load and high TNF production in HAM/TSP patients.* **(A)** Proviral load values from HTLV-1 carriers, OAB-HTLV-1-infected and HAM/TSP patients, as assayed by PCR. **(B)** PBMCs from HTLV-1 carriers, OAB-HTLV-1-infected and HAM/TSP patients were cultured for 72h, after which TNF levels were assessed by ELISA. Bars represent median values from each group. Nonparametric testing (Mann-Whitney) was used to compare among groups.
