# Supplementary figures and images for: TNF-induced metalloproteinase-9 production is associated with neurological manifestations in HTLV-1-infected individuals
Source: Front Immunol. 2022 Oct 13;13:954103. doi: 10.3389/fimmu.2022.954103 (PMC9608347; doi:10.3389/fimmu.2022.954103)

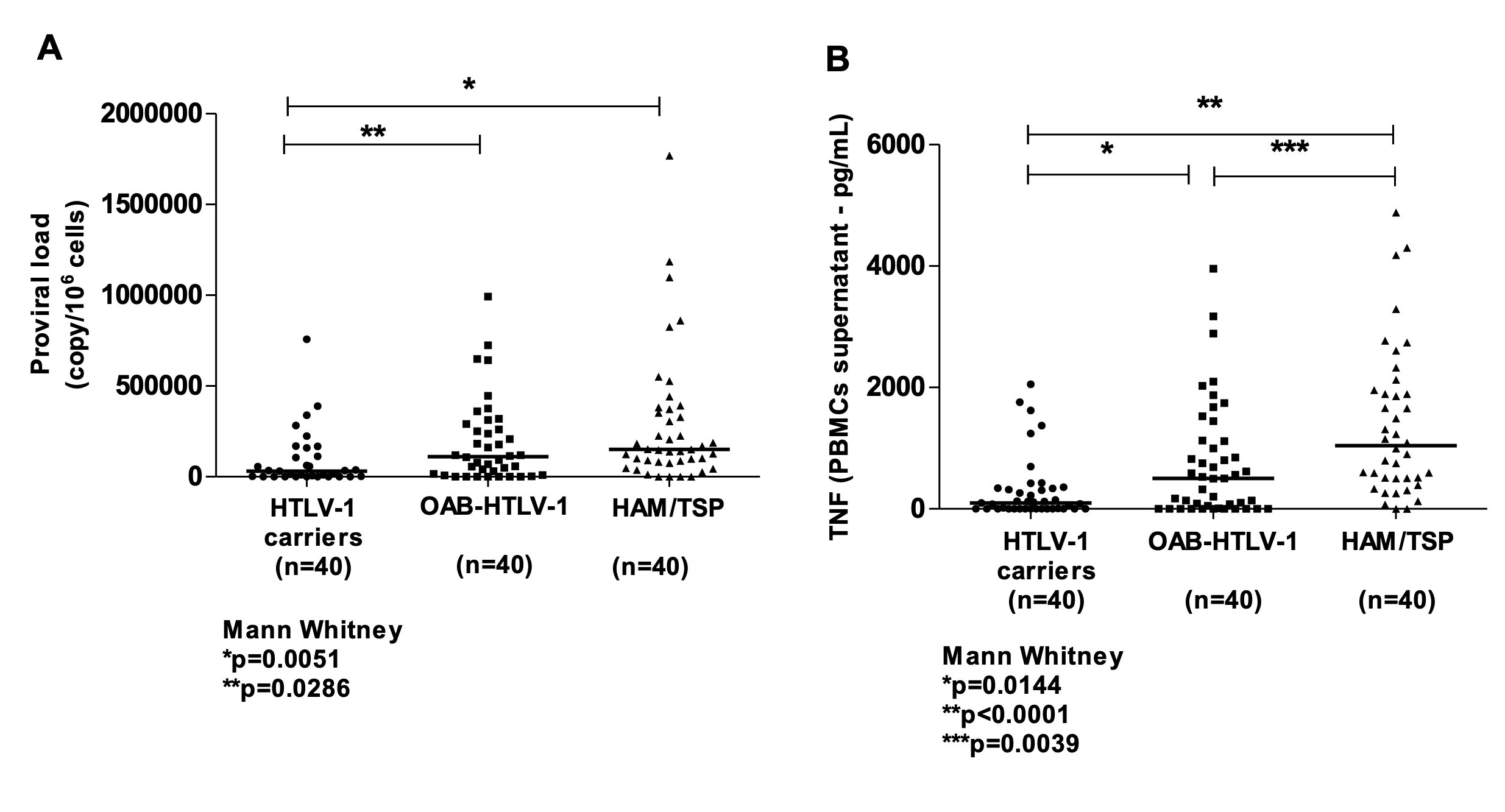

Supplement: Supplementary Figure 1 — Increased proviral load and high TNF production in HAM/TSP patients. (A) Proviral load values from HTLV-1 carriers, OAB-HTLV-1-infected and HAM/TSP patients, as assayed by PCR. (B) PBMCs from HTLV-1 carriers, OAB-HTLV-1-infected and HAM/TSP patients were cultured for 72h, after which TNF levels were assessed by ELISA. Bars represent median values from each group. Nonparametric testing (Mann-Whitney) was used to compare among groups. [file Image_1.tiff]
